# Perception of Availability, Accessibility, and Affordability of COVID-19 Vaccines and Hesitancy: A Cross-Sectional Study in India

**DOI:** 10.3390/vaccines10122009

**Published:** 2022-11-25

**Authors:** Akshay Ayappan, Bijaya Kumar Padhi, Ananthesh L., Raushan Kumar Chaudhary, Uday Venkat Mateti, Adithi Kellarai, Mazhuvanchery Kesavan Unnikrishnan, Jaclyn Drishal Dsouza, Ali Davod Parsa, Russell Kabir, Ranjit Sah

**Affiliations:** 1 Department of Pharmacy Practice, NGSM Institute of Pharmaceutical Sciences (NGSMIPS), Nitte (Deemed to be University), Mangaluru 575018, India; 2 Department of Community Medicine and School of Public Health, Postgraduate Institute of Medical Education and Research, Chandigarh 160012, India; 3 Department of General Medicine, KS Hegde Medical Academy (KSHEMA), Justice K S Hegde Charitable Hospital, Nitte (Deemed to be University), Mangaluru 575018, India; 4 School of Allied Health, Anglia Ruskin University, Chelmsford CM11SQ, UK; 5 Department of Microbiology, Institute of Medicine, Tribhuvan University Teaching Hospital, Kathmandu 44600, Nepal; 6 Department of Microbiology, Dr. D.Y. Patil Medical College, Hospital and Research Centre, Dr. D.Y. Patil Vidyapeeth, Pune 411018, India

**Keywords:** accessibility, affordability, availability, COVID-19, vaccine hesitancy

## Abstract

***Background:*** The current study aimed to identify the perceptions and issues regarding the affordability, availability, and accessibility of COVID-19 vaccination and determine the extent of vaccine hesitancy among non-vaccinated individuals. ***Methods:*** A Prospective cross-sectional study was conducted among 575 individuals for a period of six months. All the relevant information was collected using the peer-validated survey questionnaire. An independent *t*-test was applied to check the association between variables. ***Results:*** Among 575 participants, 80.8% were vaccinated, and 19.2% were non-vaccinated. Among the vaccinated, 35.1% were vaccinated in private centres and 64.9% in public health centres (PHC). In total, 32% had accessibility issues and 24.5% had availability issues. However, responders vaccinated at PHC were having more issues in comparison to other groups which was statistically significant (*p* < 0.05). Among the 163 privately vaccinated participants, 69.9% found it completely affordable. Another 26.9% and 3.1% found vaccines partly affordable and a little unaffordable. Among the 110 non-vaccinated, 38.1% were found to be vaccine-hesitant. ***Conclusions:*** Individuals vaccinated at PHC experienced issues such as long waiting times, unavailability of doses, and registration. Further, a significant level of hesitancy towards COVID-19 vaccines was observed. The safety and efficacy of COVID-19 vaccines contributed to negative attitudes.

## 1. Introduction

The Coronavirus 2019 (COVID-19) pandemic has wreaked global havoc with a total of 386,548,962 cases of infections and 5,705,754 deaths reported till 4 February 2022 [1,2]. In India, a vaccination campaign against COVID-19 was initiated on 16 January 2021 [3]. However, the skewed vaccine distribution in the wake of geopolitical disparities between industrialised and developing countries has compromised the healthcare capabilities of many major nations. India was confronting vaccine shortages due to inadequate manufacturing scale, provoking disputes between the state and the central government [4,5]. Despite the initiation of a full-fledged vaccination campaign, there is also a growing focus on vaccine hesitancy among individuals [5,6]. As a result, efforts to achieve complete vaccine coverage are being resisted, putting public health at risk from vaccine-preventable disease outbreaks. To identify the scope of this problem, our study entails identifying factors such as issues with complacency and convenience, which lead to hindrances to the vaccination drive. Complacency incorporates reasons for hesitancy, such as the safety and efficacy of vaccination, considering vaccination not necessary, and trust issues with the healthcare system [7]. Furthermore, convenience incorporates the availability, accessibility, and affordability of vaccines [8]. Vaccines are being provided free of charge by the Indian government and public health clinics. Nonetheless, vaccination costs are skyrocketing at private clinics, with no regulatory framework in place to keep them under check, which could compromise affordability for medium-to low-income individuals, thus hindering access to immunisation. Therefore, it is critical to evaluate the perception of affordability to demonstrate the feasibility of affording the vaccination [9,10].

According to the Centres for Disease Control and Prevention (CDC), immunisation is not uniformly distributed around the world, and poorer countries are failing to keep up with immunisation efforts [11]. Many states in India reported a shortage of vaccines, during the second wave of April–May 2021, possibly on account of suboptimal healthcare facilities, infrastructure, and storage facilities, particularly in suburban and rural India [12]. Rural areas are more severely underserved in healthcare than urban areas, with 63 percent of the rural requiring to travel more than 5 km due to a lack of proximal healthcare facilities [13]. Although 600 million population in India uses internet (12% of global internet user), half of its population lacks access to internet. The digital divide in India is an ongoing problem and the pandemic has definitely made it worse. Hence, it’s imperative to highlight the growing accessibility challenges, such as the digital gap and insufficient educational opportunities, as well as a shortage of skilled healthcare personnel, which needs to be addressed promptly [14,15].

This study attempts to identify the perceptions and issues regarding the affordability, availability, and accessibility of COVID-19 vaccination and determine the extent of vaccine hesitancy among non-vaccinated. The outcomes of this study will help the legislators to generate policies as per the public sentiments and to generate informed healthcare decisions to ensure a productive vaccination campaign.

## 2. Materials and Methods

### 2.1. Study Design

This prospective cross-sectional study was conducted among the inpatients as well as outpatients in a charitable hospital for six months (September 2021 to February 2022). The study population, comprising all the participants aged 18 years or older and either gender, met the inclusion criteria. Healthcare workers, individuals who had received sponsored vaccinations, and those unwilling to give consent were excluded.

### 2.2. Sample Size

The sample size was calculated based on a 95% confidence interval, 0.04 margin of error and an anticipated proportion of vaccine hesitancy of 40% [16]. The calculated sample size was found to be 575. The sample size was calculated using n Master software version -2 (Accessed on 25 October 2021: https://cmc-biostatistics.ac.in/nmaster/).

### 2.3. Data Collection

Distinct data collection forms were designed for the vaccinated and non-vaccinated populations. The first section for vaccinated individuals was about the demographic background, vaccination status, vaccine type, impact of COVID-19 on livelihood, rejected vaccination in the past, and reasons for choosing private or government centres. The second section generated a response to the affordability of vaccines on a four-point scale (unaffordable to completely affordable), along with reasons for unaffordability. The third section contained a close-ended question to determine issues with availability and accessibility, with the reasons for the same. Further inquiries were made regarding issues encountered during the registration process. The final section evaluated the outcomes of vaccination if an individual contracted COVID-19 recently and was hospitalised. This section also collected details about Adverse Events Following Immunisation (AEFI).

All the non-vaccinated participants were given a questionnaire to assess whether or not they would be likely to get vaccinated when the vaccine was available at an affordable, well-equipped sanitary healthcare centre. The options for vaccine hesitancy ranged from very likely to rather likely to neither likely nor unlikely. In addition, willingness to pay (WTP) for a vaccine was obtained. The Kuppuswamy scale was used to evaluate the socioeconomic status of respondents.

A panel of experts validated the questionnaires for relevance and clarity. Following minor modifications, the questionnaire was put to a pilot test on a randomly selected group who did not participate in the main study. The pilot trial was voluntary. Anonymity was guaranteed for individual responses.

### 2.4. Statistical Analysis

The data were entered into a Microsoft Excel spreadsheet, reviewed twice for accuracy, and analysed using the Statistical Package for Social Science (SPSS) 26.0 for Windows. Frequency and percentage were used to calculate and describe the descriptive data. An independent *t*-test was applied to check for an association between variables. Statistical significance was defined as *p* < 0.05.

## 3. Results

A total of 575 participated in the study, of whom (n = 465, 80.8%) were vaccinated and (n = 110, 19.2%) were non-vaccinated. Among the vaccinated, (n = 163, 35.1%) were vaccinated in private centres and (n = 302, 64.9%) in government lead public health centres (PHC). The major reasons for choosing private facilities were regular better availability of vaccines (n = 77, 47.3%), and better hygienic services (n = 49, 30%) whereas the availability of vaccines free of cost (n = 257, 85.5%) was the main reason for choosing primary healthcare centres. Among the vaccinated, the majority were fully vaccinated 66.8%, while 33.1% were partially vaccinated. The mean age of the participants in the study was found to be 52.74 ± 14.19. Among the vaccinated and non-vaccinated, 56.6% and 63.6% were males, respectively. The majority (34.8%) of the vaccinated had a master’s degree, while 13.1% had less than a high school degree. In contrast, the majority (72.7%) of the non-vaccinated had less than a high school degree, and only 2.7% had a master’s degree (*p* < 0.01). Among the vaccinated, 39.7% of those who received vaccinations were from the lower middle class, 29.9% were from the upper middle class, and only 1.3% were from the lower class. Similarly, 37.2% of the non-vaccinated belonged to the lower middle class, followed by 33.6% upper lower class, and only 4.5% represented the upper class (*p* < 0.01). The impact on livelihood was severe among 68.1% of the non-vaccinated compared to the vaccinated individuals (28.6%), (*p* < 0.001). Among the non-vaccinated, the majority 69% had recently contracted COVID, whereas only 35% of the vaccinated individuals were affected. The hospitalisation rate among the non-vaccinated was higher at 35.5% compared to the 14.7% vaccinated individuals, (*p* < 0.05). The general characteristics of study participants are further summarised in Table 1.

Overall, (n = 325, 75.6%) of the total vaccinated population received covishield, (n = 109, 23.4%) received covaxin, and (n = 4, 0.8%) received sputnik. When asked what influenced the patient to get a vaccination, the majority (n = 312, 67%) were self-influenced, while the remaining (n = 85, 18.3%) had government influence. Overall, (n = 140, 30.1%) of the participants had issues with the registration process, out of which 70% had complaints of service issues and other malware glitches. In addition, 71.8% of vaccinated responders complained about adverse events post-vaccination. Fatigue (21.4%) and pain (19.7%) were the most common symptoms, whereas fewer reported chills (13.9%) and swelling (7.8%) at the site of injection (Table 2).

Among the 163 individuals vaccinated in private centres, (n = 114, 69.9%) found it completely affordable. Another (n = 44, 26.9%) and (n = 5, 3.1%) found vaccines partly affordable and a little unaffordable, respectively. Reasons for unaffordability were 44.8% loss of jobs, 38.7% liabilities, and 16.3% budgetary concerns to meet healthcare expenses.

Issues with accessibility were most prevalent among PHC vaccinated responders (n = 145, 48%) compared to (n = 3, 1.8%) of private vaccinated responders and (n = 36, 37.2%) non-vaccinated individuals. Lack of knowledge to register at 31.0%, long waiting time at 26.2%, and fewer healthcare workers at 20.7% were the most common accessibility issues faced by PHC responders. Overall, considering those who were vaccinated at PHC, private centres, and non-vaccinated individuals, prolonged waiting time was the major accessibility constraint. Likewise, availability issues were most prominent among PHC-vaccinated responders (*p* < 0.01), compared to private-vaccinated responders and non-vaccinated. Among PHC A major concern was a lack of available doses, followed by a lack of storage facilities and a poor healthcare setting and infrastructure. Table 3 summarises accessibility and availability issues among the participants.

Among non-vaccinated, the majority (n = 32, 29%) were very likely to get vaccinated, but (n = 24, 21.8%) and (n =18, 16.3%) were rather unlikely and very unlikely to get vaccinated, contributing to (n = 42, 38.1%) vaccine hesitancy. Major reasons for vaccine hesitancy were concerns over the safety and efficacy of vaccines at (n = 37, 88%), feelings of non-essentiality at (n = 15, 35.7%), and a lesser reason was the presence of medical conditions in which vaccines have not yet been tested at (n = 6,15%), respectively. The remaining non-vaccinated had (n = 36, 37.2%) accessibility issues and (n = 32, 29%) availability issues. A majority of 50% were WTP 200–400 INR, 27.2% were WTP 400–600 INR, and only 6.3% of respondents were WTP > 800 INR per head for the vaccination. Table 4 summarises vaccine hesitancy questions and reasons for the same.

## 4. Discussion

Among the accessibility issues, the majority of PHC respondents (31%) lacked knowledge on how to enrol for vaccination. Similarly, a Boston Consulting Group survey found that 63% of people in rural areas lacked the knowledge to register for the CoWIN App [17] Which is justified by India’s digital divide between rural and urban families, Digital infrastructure, internet connectivity, literacy, and even electricity availability is inadequate and inconsistent in several parts of India [18,19].

An interview-based study pointed out that waiting times among public sector facilities could be longer for the same ailment than private sector facilities [19]. People seeking vaccine in several parts of the country reported long waiting times and being turned away because doses had not arrived at the centres, which lead to reluctance towards the vaccination drive [20,21]. Similarly, our study revealed that long waiting time was the second most common accessibility issue among 26.2% of PHC responders. This could be due high volume of patients served in the PHC, hence resulting in prolonged waiting periods. While private hospitals have shorter waiting periods [22].

Despite the fact that the number of healthcare institutions has increased over the decade, there are significant workforce shortages. As per our findings, 20.7% of PHC respondents identified a lack of healthcare workers and a 15.2% lower number of nearby healthcare centres as one of the significant contributors to the accessibility issue. A recent WHO report, reveals that India requires at least 1.8 million doctors, nurses, and midwives to meet the minimum threshold of 44.5 health workers per 10,000 populations by 2030 [23]. Furthermore, PHC use has declined substantially over the previous two decades, with only 32% of urban Indians utilising them currently, down from 43% in 1995–1996 [24]. Similarly, a report by Sharma et al., revealed an unequal distribution of healthcare centres, with one government hospital bed for every 1833 people, compared to 2336 people a decade ago [25].

Among the availability issues encountered by the PHC, the lack of availability of doses was a major concern 47.9%, followed by the lack of a storage facility 41.8%, and lastly, a poor healthcare setting 10.2% which could be justified by the government data, in June 2021, only 30 million people had received a second dose of vaccination, which contributes to only 2% of the entire 1.3 billion population [26]. Correspondingly, as per May 2021 statistics, India’s per capita vaccination rate was around 5%, compared to 60% in Israel, 46% in the United Kingdom, and 32% in the United States [27]. However, following the second wave, there was a drop in the number of cases, providing calibration time for vaccine manufacturers to meet the exceeding demand, and thus the availability concern was gradually reduced.

Our study showed 30.1% of vaccinated faced issues with registration. Out of which, the majority of complaints are about issues with servers, software glitches, and internet connectivity. Other concerns raised were delays in obtaining One-time Password (OTP) and the Adhaar card not being linked to a phone number. Due to these shortcomings, vaccinators have been forced to adopt simpler distribution strategies [28]. Inconsistencies and issues in registration and slot allotments started to surface slowly after its launch. As of now, more productive improvements and corrections have been made, and the government intends to repurpose the Co-WIN platform for India’s Universal Immunisation Program [29].

The majority of the privately vaccinated individuals 69.9% found the pricing affordable, followed by 26.9% partially unaffordable and 3% completely unaffordable. Sources revealed that the median price at the private centres sampled was 75% of the average monthly expenditure of the poorest urban household and 29% of the average monthly expenditure of the middle class [30]. However, the reasons for accepting paid vaccination despite high soaring prices were improved services, availability, and sanitary conditions provided by private centres. The government-run PHC faces a significant challenge in meeting the expected standards and levy of private centres.

Currently, vaccine hesitancy worldwide as well as in India is on a declining trend. Notably, 44.5% of the non-vaccinated were (very or rather) likely to get vaccinated, 17.2% were neutral (neither likely nor unlikely), and 38.1% were (very or rather) unlikely to get vaccinated. This is in concordance with another study by Mascherini et al. [31]. Hence, contributing to the (38.1%) of non-vaccinated individuals in the vaccine hesitancy group, a similar trend was observed in the study by Iwu et al., in which the prevalence of hesitancy was 35.4% [32]. Concerns about vaccine safety and efficacy accounted for 88% of vaccine hesitancy. It’s in correspondence to another study by Syed Alwi et al., 84.7% contributed to safety concerns [33]. The secondary reason was a feeling of non-essentiality to getting vaccinated at 35.7%, in concordance with studies conducted by Jaffe et al., in which 30.0% of the individuals reported not believing it was necessary to get vaccinated [34]. Moreover, 24.4% of people in our studies had trust issues with vaccination drives and the overall healthcare system, which paralleled studies by Biswas et al., who reported 22% of the same [35]. Other reasons for hesitancy were fear of needles and the presence of comorbid conditions in which vaccines have not yet been tested, these findings are in concordance with the findings by Freeman et al., and Jaffe et al. [34,36]. It’s noteworthy that aside from vaccine hesitancy, other non-vaccinated preferred not to be immunised due to 32.7% accessibility and 29.0% availability issues. Similarly, studies by Khairat et al. reported other major predictors for vaccine hesitancy were lack of access and vaccine availability issues [37].

The majority (72.7%) were in the “less than high school” category, followed by 12.8% high school, 11.8% undergraduate, and 2.7% PG diploma/master’s level of education. In addition, a study by Khairat et al., reported concordant findings in which more than half (65%) of non-vaccinated adults with vaccine hesitancy had a high school or less than a high school education [37]. Another survey revealed that 28.8% of participants rejected the vaccine outright, which is strongly associated with lower educational level, and lower perceptions of COVID-19 severity [38].

Under accessibility issues, the majority of non-vaccinated complain of long waiting times 58.3%, followed by a lower number of healthcare centres 27.7%, and a lower number of qualified healthcare workers of 8.3%. Likewise, another study by Cherian et al. reported concordant results, in which non-vaccinated had issues with long waiting times 16.8% had a prominent higher position, followed by a lower number of proximal healthcare centres 9.9% and 4.9% lack of qualified healthcare workers/bad experience with vaccinators [39]. The severe impact on livelihood was more significant among the non-vaccinated participants, 68.1%. The majority of non-vaccinated belong to rural and lower socioeconomic statuses, making them more susceptible to economic distress. This could be due to multiple confounding variables such as socioeconomic indicators (such as teleworking and income per capita), restricted access to public health information, healthcare, and cleanliness. Furthermore, the national lockdown aggravated the impact on livelihood among the rural population, which resulted in a drop in annual sales and revenue for the country’s workforce [40].

The majority of non-vaccinated were WTP at a lower range of 200–400 INR, 55 (50%), which is contrasting to the report by Goruntla et al., in which the majority of respondents were willing to pay 500–1000 INR [41]. This implies that private, high-quality healthcare services may be out of reach for average Indians, even for COVID-19 immunisation. The minimum price of the vaccination is ($10.5), 819 INR per dosage per person, which is approximately 81% of pre-crisis monthly family income as computed from the most recent available National Sample Survey (NSS) round from 2017 to 2018 [30].

It is worth noting that the study was conducted in an area with a high HDI score (Human Development Index) of 0.83, which is exceptionally high in contrast to other states in India [42]. Despite a high development index, vaccine hesitancy and the 3A’s (availability, affordability, and accessibility) have seen serious challenges. There is the potential for conditions to be more severe in other states. Therefore, it’s imperative to establish the following recommendations by the policymakers to reduce the inconvenience regarding vaccination among the general public and to taper vaccine hesitancy among the non-vaccinated.

The issue of vaccine shortages could be temporarily alleviated by employing the vaccine dose fractionalisation method. It is a method in which a fraction of the standard dose is administered via the same route [43]. This method was proven to be effective in a yellow fever vaccine drive in Angola and the Democratic Republic of the Congo (2015), where patients were given one-fifth of the regular dose [28]. Better information on vaccine dosage, incoming supplies, and consistent allotment would enable better appointment planning and help to mitigate long wait times in healthcare facilities. The establishment of a dedicated call line for the general public to call if they require assistance with the registration procedure will aid in overcoming the lack of expertise in the registration process, and developing a relationship with local pharmacy shops that can schedule a vaccine appointment could ease the registration process, particularly for the elderly. In addition, training additional paramedics to offer basic life support and injectable will relieve and tackle the workforce shortage. As many individuals do not have access to the news or social media, it is critical to research alternative means of communication in order to eliminate vaccine myths. To begin, conducting surveys among health practitioners and community members will aid in gaining a better knowledge of local opinions and the extent of misinformation, which can then be used to target future messaging activities. Second, trustworthy community voices should serve as message carriers to the broader public, informing them about the safety and efficacy of vaccinations and urging them to be vaccinated. 

However, the study’s conclusions must be evaluated in consideration of certain limitations. Since it is a single-centric cross-sectional survey, the data are restricted to a specific sub-region and cannot be considered a nationwide scenario. As the findings of this study are dependent on the respondents’ opinions and knowledge, they are vulnerable to response bias. When comparing vaccine hesitancy with other studies, it’s imperative to note that the same techniques have been employed by other research to express the likelihood of taking COVID-19 vaccination. Furthermore, the sample size for the study was calculated by taking 40% vaccine hesitancy which might affect the power and result of the study as 20% vaccine hesitancy has been also reported.

## 5. Conclusions

We conclude that availability and accessibility issues were prevalent among individuals vaccinated at primary care centres, with long waiting times, unavailability of doses, and registration issues being the most commonly reported issues. Furthermore, we found a significant level of hesitancy towards COVID-19 vaccines. Individuals with negative attitudes were majorly concerned about the safety and efficacy of COVID-19 vaccines. Thus, the implementation of proposed recommendations by policymakers could achieve better vaccine convenience and complacency.

## Figures and Tables

**Table 1:** General Characteristics of Participants in the Study.

Parameters	Vaccinated n = 465	Non-Vaccinated n = 110	Overall n = 575	*p*-Value
**Age (Mean ± SD)**	54.99 ± 13.06	43.20 ± 14.85	52.74 ± 14.19	
**Gender**				
Male	263 (56.6)	70 (63.6)	333 (57.9)	0.373
Female	202 (43.4)	40 (36.4)	242 (42.1)	
**Education**				
Less than high school	61 (13.1)	80 (72.7)	141 (24.52)	<0.01 *
High school	100 (21.5)	14 (12.7)	114 (19.82)	
Under graduation	142 (30.5)	13 (11.8)	155 (26.95)	
Diploma/Post Graduation	162 (34.8)	3 (2.7)	165 (28.69)	
**Socioeconomic status**				
Upper class	110 (23.6)	5 (4.5)	115 (20)	
Upper middle class	139 (29.9)	16 (14.5)	155 (26.9)	<0.01 *
Lower middle class	185 (39.7)	41 (37.2)	226 (39.3)	
Upper lower class	25 (5.3)	37 (33.6)	62 (10.7)	
Lower class	6 (1.3)	11 (10.2)	17 (3.01)	
**Rejected vaccination in the past**				
Yes	106 (22.8)	65 (59.0)	171 (29.8)	0.934
No	359 (77.2)	45 (40.9)	404 (70.2)	
**Impact on livelihood**				
Mild	135 (29.0)	9 (8.1)	144 (25.0)	
Moderate	197 (42.3)	26 (23.6)	223 (38.7)	<0.01 *
Severe	133 (28.6)	75 (68.1)	208 (36.1)	
**Recently contracted COVID-19**				
Yes	163 (35.0)	76 (69.0)	239 (41.5)	<0.01 *
No	302 (64.9)	34 (30.9)	336 (58.4)	
**A. Hospitalised due to COVID-19 Infection**				
Yes	24 (14.7)	27 (35.5)	51 (21.3)	
No	139 (85.3)	49 (64.5)	188 (78.7)	0.07

* Statistically Significant.

**Table 2:** Specific characteristics among vaccinated participants.

Parameters	Vaccinated n = 465
**Vaccination status**	
Partially vaccinated	154 (33.1)
Fully vaccinated	311 (66.8)
**Vaccination Type**	
COVISHIELD	352 (75.6)
COVAXIN	109 (23.4)
SPUTNIK	4 (0.8)
**Issues with registration process?**	
Yes	140 (30.1)
No	325 (69.9)
**A. Type of issues faced**	
Adhaar not linked to mobile number	17 (12.2)
Delay in receiving One-time Password	25 (17.8)
Others (server issues, glitches)	98 (70)
**Adverse events following immunisation**	
Yes	334 (71.8)
No	131 (28.2)
**Type of Adverse Events**	
Pain	283 (19.7)
Fever	281 (19.5)
Swelling	113 (7.8)
Headache	256 (17.7)
Fatigue	308 (21.4)
Chills	199 (13.9)

**Table 3:** Accessibility and availability issues among the participants.

Parameters	Private Centres n = 163	PHC n = 302	Non-Vaccinated n = 110	Overall n = 575	*p*-Value
**Accessibility issues?**					
Yes	3 (1.8)	145 (48)	36 (32.7)	184 (32)	<0.01 *
No	160 (98.2)	157 (51.9)	74 (67.2)	391 (68)	
**A. Types of accessibility issues**					
Less healthcare centre	1 (33.3)	22 (15.2)	10 (27.7)	33 (17.9)	
Less number of healthcare workers	0	30 (20.7)	3 (8.3)	33 (17.9)	0.129
Lack of knowledge to register	1 (33.3)	45 (31.0)	2 (5.5)	48 (26.0)	
Commuting issues	0	10 (6.8)	0 (0)	10 (5.4)	
Long waiting time	1 (33.3)	38 (26.2)	21 (58.3)	60 (32.6)	
**Availability issues?**					
Yes	11 (6.7)	98 (32.4)	32 (29.0)	141 (24.5)	<0.01 *
No	152 (93.2)	204 (67.5)	78 (70.9)	434 (75.5)	
**A. Types of availability issues**					
No available doses	6 (54.5)	47 (47.9)	19 (59.3)	72 (51.0)	<0.01 *
Lack of storage facility	4 (36.4)	41 (41.8)	13 (40.6)	58 (41.1)	
Poor health settings	1 (9)	10 (10.2)	0 (0)	11 (7.8)	

* Statistically Significant.

**Table 4:** Vaccine hesitancy among non-vaccinated participants.

Parameters	Non-Vaccinated n = 110
**How likely is it that you will get vaccinated even when the vaccine is available to you at an affordable, well-equipped, and sanitary healthcare facility?**	
Very likely	32 (29%)
Rather likely	17 (15.5%)
Neither likely nor unlikely	19 (17.2%)
Rather unlikely	24 (21.8%)
Very unlikely	18 (16.3%)
**Vaccine hesitancy (Rather unlikely + Very unlikely)**	42 (38.1%)
**Reasons:**	
Concerns over safety and efficacy of vaccine	37 (88.0%)
Feeling of non-essentiality to get vaccinated	15 (35.7%)
Trust issues with vaccination drive or healthcare system	11 (24.4%)
Fear of needles	9 (20%)
Presence of Medical condition in which vaccines are not yet tested	6 (15%)
**Willingness to pay in INR:**	
200–400	55 (50.0)
401–600	30 (27.2)
601–800	18 (16.3)
>800	7 (6.3)

INR—Indian rupees.

## Data Availability

Not applicable.
